# The New York Institute of Stomatology

**Published:** 1901-09

**Authors:** 

**Affiliations:** The New York Institute of Stomatology


### THE NEW YORK INSTITUTE OF STOMATOLOGY.

A MEETING of the Institute was held at the office of Dr. J. Adams Bishop, 30 West Forty-eighth Street, on Tuesday evening, May 8, 1901, the President, Dr. J. Morgan Howe, in the chair.

The minutes of the last meeting were read and approved.

### COMMUNICATIONS ON THEORY AND PRACTICE.

*Dr. George S. Allan.*—Shortly after Dr. Frederic Bogue read his paper there was a synopsis of it published in the *Dental Re-*

*view.* Almost coincident with the appearance of this in the *Review,* I received a letter from Dr. Miller asking me if I would bring before the Odontological Society his views on this subject as published in the *Independent Practitioner* of 1883, pages 632, 633, and in his book, "Micro-organisms of the Human Mouth," page 196.

After writing to Dr. Miller I received a letter from him stating that he had supposed the paper had been read before the Odontological Society, and asking me if I would bring the matter of his paper before the Institute of Stomatology.

In the article published in the *Independent* Dr. Miller mentions having broken up a number of teeth free from caries and producing decay artificially in a mixture of bread and saliva. The phenomenon of white decay was produced with greatest accuracy. Various stains, such as tobacco, etc., were also used, and gave the varying degrees of color noted in decay in the mouth. Where enamel was hard and dense, without crack or blemish, it had not even lost its lustre. He mentions that this experiment shows what a vast difference the structure (density) of the tooth makes in its resistance to decalcification, and that it offers an explanation why all teeth do not decay alike; that a dense tooth covered with enamel, intact at every point, would probably resist for years the action of an acid saliva to which a soft defective tooth would succumb in a few weeks; also the fact, established by the above experiment, that the rapidity with which tooth-tissue is acted upon by the acid of tooth caries is inversely proportional to some multiple of the density of the tooth, he considers to be of greatest importance, as furnishing a key to the solution of the question of the great difference in the liability of teeth in different persons, or of different teeth in the same person, to decay.

In his book, "Micro-organisms of the Human Mouth," Dr. Miller speaks of the production of artificial decay by means of bread and saliva, and says that it cannot be distinguished from the decay occurring in the mouth.

*Dr. S. E. Davenport.*—I would like to ask Dr. Allan if Dr. Miller in his letter gave us any hope that he would give the Institute a paper upon this subject, and also if Dr. Miller expressed his approbation of Dr. Frederic L. Bogue's paper?

*Dr. Allan.*—I regret very much that I did not bring Dr. Miller's letter. Dr. Miller does not say distinctly that he will prepare a paper, but he intimates that he has something in preparation and also that when it is ready the Institute may have it. From his letter it is very evident that his views coincide entirely with the results of Dr. Frederic L. Bogue's experiments. I am very sorry to say that I have learned indirectly that Dr. Miller is not in the very best of health, and undoubtedly he does not care to rush into print until he is fully prepared. I shall write Dr. Miller at once, and hope to have something to communicate from him later.

There is one little point that I would like to bring before the Institute, and that is a method which I have of filling roots with oxyphosphate. I have heard it advocated recently to fill these roots with a syringe, the fine point of which will go to the bottom of the canal, and as the thin filling-material is forced into the root the syringe point is gradually withdrawn. This would be an excellent way, no doubt, but I have never seen a syringe point that would go to the bottom of a root-canal. My method is to wrap a little cotton round a stiff probe and with this work the oxyphosphate down into the root. I can do this about as quickly as one could syringe it into the canal, provided he could get a syringe that would go into a root-canal. I do not see but what this meets the requirements. I mix the oxyphosphate rather soft.

*Dr. E. A. Bogue.*—Some years ago I used ethyl chloride on a certain occasion to obtund sensitiveness in a lower molar, and as my victim is now in the room I wish to say a few words regarding ethyl chloride for this purpose. Dr. Flagg says we talk about our successes and not about our failures. It is one of my failures which I will mention. At that time I did not understand the possibilities of ethyl chloride, and I used it to remove the pulp. Both the patient and the operator had a dreadful time. Since then I have tried it again and again, and I have been quite successful in obtunding sensitive cavities sufficiently to excavate without pain in nearly all the upper teeth and in the lower front teeth, but not very successfully the lower posterior teeth. I had a very timid little girl in my chair to-day, thirteen or fourteen years of age. I excavated four very sensitive cavities, and I think there were just four tears. I use for this purpose the ethyl chloride, which comes in tubes with a bent nozzle, over which there is a cap with a spring handle, which can be lifted at will. I put quite a large piece of cotton loosely in the cavity, and apply and remove alternately the stream of ethyl chloride to this cotton once in two or three seconds.

*The President.*—Is the bottle referred to the one in which the chloride is furnished?

*Dr. Bogue.*—Yes.

*Dr. Davenport.*—The secret of Dr. Bogue's present success with ethyl chloride is probably due to his method of applying it. The constant stream used by most operators causes such rapid evaporation that the pain is intense. If the stream is stopped occasionally, the benumbing influence of the drug may have some effect.

*Dr. Bogue.*—Of course, after the cavity has become insensible to the stream of ethyl chloride the cotton can be removed and the stream thrown directly into the cavity. This, however, should not be done for more than two or three seconds.

*The President.*—Does Dr. Bogue think this method superior to cataphoresis?

*Dr. Bogue.*—Mr. President, cataphoresis is never mentioned up our way. It is so far back that I have forgotten all about it. Intense heat and intense cold are both benumbing influences. Both will anæsthetize to a degree. Heat will cause the patient just about as much pain as the chloride of ethyl. After a few seconds (ten or twelve) the pain will cease. After this point the difference is, that the hot air has to be constantly applied.

The chloride of ethyl can be applied directly upon the surface of a tooth, benumbing the whole tooth, thus enabling the operator to remove an amalgam filling. A gentleman came to me some years ago with an upper cuspid tooth which had become so sensitive that he could not endure it any longer. I told him to stop in next morning early and I would remove the pulp and fill the cavity for him. He did not believe it. He came in. There was no cavity in the tooth. By means of the chloride of ethyl I thoroughly anæsthetized the tooth, drilled into it, and removed the pulp; then filled the tooth and sent him away before nine o'clock. He said it was done absolutely without pain. However, this is only one of the various methods of relieving pain.

*Dr. H. W. Gillett.*—There are two questions that I would like to ask. One is whether there is any injury to the pulp of the tooth by the use of the chloride of ethyl? and the other, if there is any pain on the tooth regaining sensation,—anything corresponding to the pains in the fingers as they regain sensation after being severely chilled?

*Dr. Bogue.*—I have never seen any injury to the pulp when the process is not carried beyond what is necessary to obtund the sensation of the dentine. I have noticed a little pain on recovery a few times. I remember one case where it was some time before the patient and I could come to terms regarding the payment of his bill, because of continued sensation in a cavity which extended under the gum.

Dr. George F. Eames's paper on "The Treatment of Certain Oral Expressions commonly designated as Pyorrhœa Alveolaris" was then read.

(For Dr. Eames's paper, see page 605.)

*Dr. C. O. Kimball.*—Dr. Briggs, of Boston, not being able to be present this evening has forwarded his discussion, which I will read.

"I am very sorry not to be with the Institute in person, for written communications not read by their authors are apt to fall flat, and were I there I should receive some inspiration from the presence and remarks of the other members. There is little or nothing left for me to add to what has already been said on this subject, and the only excuse for saying anything lies in the fact that in spite of all that has been said and written there are still many practitioners of good standing who practically tell their patients that there is nothing for the relief or cure of pyorrhœa alveolaris. The fact is that we do relieve and do cure a very large proportion of the cases that present themselves. Let us briefly review what we know about this disease. First, its etiology. It may be due to local or constitutional conditions, alone or combined. Local treatment undoubtedly helps all cases, but it is equally true that many cases could be much better helped did we know the constitutional cause and give treatment accordingly. We know some cases to be of syphilitic origin, and suspect many, many others. In these cases when antisyphilitic medicines are given in connection with local treatment the results are magical.

"Auto-intoxication is another cause which when diagnosed may be treated by dieting and eliminants with satisfactory results. As time goes on we shall unquestionably add to our list of constitutional causes, and, with increased ability to diagnose them, more rapid and satisfactory results from treatment will follow.

"We must not forget the influence of metallic poisons in producing pyorrhœa alveolaris, whether absorbed accidentally or as the result of drugging. But, however much we may add to our knowledge of constitutional causes and constitutional treatment, there still remains the fact that many cases are due to local causes and call for local treatment only; also that all cases from local or constitutional causes are materially helped and sometimes cured by local treatment alone. I will not weary you with discussion of the pathology and diagnosis of this disease, but will pass on to the question of treatment, local treatment.

"For convenience let us divide the cases into four classes,—(1) the hopeless; (2) the very bad; (3) the bad; (4) the curable.

"In the first-mentioned, or hopeless, class we find the tooth extremely loose, the pockets very extensive, reaching the apex of the root at various points, and containing pabulum as well as pus; the alveolar process practically absorbed, the pulp dead, and pressure on the tooth causing pain. The treatment in these cases is extraction.

"In the next class, or 'very bad,' we find a tooth quite loose; deep pockets extending at one or more points to the apex of the root; pulp in many cases dead; nodules of tartar difficult of access (as between roots of upper molars); discomfort on pressure. In these cases extraction and replantation offer almost the only sure means for the removal of the nodules. Where, however, they can be reached one need not extract, but the pulp should be destroyed and the root filled. These teeth should receive steady reliable support from some form of splint, which may be worn six weeks or a lifetime, as the case may require. Prognosis, unfavorable.

"The next class, or 'bad,' includes the teeth about which the pockets are not deep enough to reach the apex, but which are more or less loose and irritable and with the tartar scales often in inaccessible regions. If this latter condition obtains, extraction and replantation are in order. In any event a permanent or temporary splint should be adjusted, the pulp removed, and the root filled. In my opinion this is a *sine qua non* in the treatment of this class of cases. Prognosis, favorable.

"The last class, or so-called curable cases, are the only cases we ever ought to see, and will ever see when the profession become educated in the diagnosis and treatment of the disease. When due to local conditions alone it takes about five years to produce the third class, or 'bad' cases, and every patient who cares for his teeth at all comes under observation at less intervals than that, and the patients in whom constitutional disturbances are at work are pretty sure to come under observation while they, the constitutional disturbances, are operating.

"These cases call for the treatment that the other cases previously mentioned demand, and in addition, the treatment which should be given to all other teeth not already affected, as a means of prophylaxis. This treatment can be summed up in the one word 'cleanliness.' Alas! how few of us seem to know what cleanliness means, or the methods of producing it! Dr. Smith, with his painstaking polishing of each tooth individually, and Hare, with his sterilizing, only emphasize a principle which if once thoroughly inculcated could be worked out by every practitioner without following the special instructions of any one man.

"There are numberless instruments for removing the tartar. I do not suggest any particular one; I only say, *get the tartar off*. There are many antiseptics. Take your choice, but make the pocket aseptic. There are enough stimulating astringents. Use one, and give tone to the gum while favoring new growth. There are solvents for viscid fluids (I might almost call them greasy) which surround the neck of teeth with pyorrhœa, and there are variations in the technique of polishing those necks to make them self-cleansing and allow the gum to lie close with a thin clear margin. But this is not an A, B, C class, and I will not try to teach methods, only begging you to keep constantly in mind the underlying principles, and reminding you that, with the idiosyncrasies of patients, an astringent is not always an astringent and no one drug is ever going to fit all your cases."

*Dr. Charles F. Allan.*—Mr. Chairman and gentlemen, great merit is usually accompanied with considerable modesty, as we have seen in the case of the essayist to-night. Certainly a more practical and helpful paper could not have been given us. It is possible that, as regarding the etiology of the disease, I might in some ways differ with him, but in the treatment I am certainly at one with him. He speaks of his instruments. According to my ideas of this affection, instrumentation is most necessary. I think it was Dr. Mitchell who, in a paper recently published in the *Dental Cosmos,* spoke of this disease as hereditary diathesis plus intemperate feeding. What he means by intemperate feeding is the improper assimilation of the nourishment taken into the system. Now, in describing the etiology of pyorrhœa, I would say "*hereditary diathesis,* plus advancing years, plus intemperate feeding." By this I mean to say that this is a condition and not a disease; that it is a condition mainly dependent upon advancing years; that as occasionally we see very young persons lose their hair, or young persons with gray hair, so do we occasionally find this condition in young people; it is, however, simply an indication of advancing years, helped on by heredity and faulty metabolism. The teeth are dermal in origin, the same as the hair, and why should not their environment be affected in the same way? I think, then, we should bear in mind in the treatment of pyorrhœa that we are treating a condition and not a disease. In the treatment the removal of every particle of calcic deposit is the first essential. The essayist has spoken of dipping his instruments in a solution of carbolic acid, which is a very good thing. I find that some patients object very strongly to the smell of carbolic. I have a habit, when cleansing teeth, of holding my instrument frequently under a stream of running water. He spoke of using compressed air to rinse out under pressure. Compressed air is a very useful adjunct to every office. My attention has recently been called to a syringe, the platinum points of which are removable, so that they can be thoroughly sterilized by heat. He spoke about using a rubber brush to massage the gums. This undoubtedly would be a very good thing unless it means the giving up of the bristle brush. I think both should be used. I thought, on looking over the synopsis of the paper which was sent me, that it would treat more on the etiology of the conditions, but I am very glad that it has taken this more practical turn. Whether every one here believes Dr. Eames's idea that it is a disease, or takes up with my suggestion that it is a condition, certainly every one must agree with Dr. Eames's practical suggestions regarding treatment. I wish to thank Dr. Eames for his helpful paper.

*Dr. Gillett.*—It has been puzzling me for some time to understand why the chairman of the Executive Committee selected me to say anything on this subject. If there is any one subject more than another that I know nothing about it is pyorrhœa. I wish to compliment the essayist upon his valuable paper. I have gained from it many ideas. I have not been so fortunate, or so unfortunate, as you may choose to call it, as to have many marked cases of pyorrhœa in my practice. What little experience I have had has been along the line of preventive treatment, and that of course has been the keynote of what has been said to-night. It seems to me that the prevention of a large proportion of the cases of so-called pyorrhœa is a simple matter. Pyorrhœa being a filth disease, its prevention is largely a matter of cleanliness. A large percentage of all the cases can be traced directly to neglect either from the patient or the operator. One of the things that I was in the habit of saying to students was to the effect that if pyorrhœa occurred in the mouths of patients who grew up under their care they were to blame: that either they failed to do their duty as operators, or that they failed to develop sufficient influence to compel proper care on the part of the patients. I do not feel like saying that to-night; I do not feel like saying that I fully endorse it, but nevertheless there is a good deal of truth in it. Working along that line, it has been my theory that the cleansing of the teeth is one of the most important operations that I do in my office, and the operation that is always to be done by my own hand. I do not believe it is the operation which can be left for the assistant, and I try to impress the importance of it upon my patients; neither is it an operation which is to be charged at a reduced rate, for I believe I do my patients more good by this operation than almost any other that I do for them. Along that line comes a word or two regarding instruments. Of course, we all have our own pet instruments. I think the reason why some men fail in this operation is because they have not in their cabinets the proper instruments for this work. Instruments which are ordinarily put upon the market by the supply-houses are not at all suited for this work. They are clumsy and unsatisfactory. I believe, as a general thing, the instruments that are used for cleansing are not delicate nor fine enough. I have here a few selected from my own cabinet which I will present for your inspection. Dr. L. L. Buckland has said that if the mouth be kept as clean as the face it will last as long and look as well. I do not mean to say that I believe all cases of pyorrhœa can be eliminated in this way. I recognize the fact that there are cases dependent upon systemic conditions, but I feel that if our young men were more thoroughly impressed with the necessity of proper and regular cleansing of the teeth of their patients this condition would not develop so frequently.

*Dr. George S. Allan.*—The question arises, What is pyorrhœa alveolaris? I have never seen a definition that I thought would hold water, and if I am asked the question I have to practically say that I do not know. Of course, the term itself means a flow of pus from the alveolus, but only a minority of the cases that come to us have this flow of pus. Again we have cases where there is pus and no deposit, and again cases where there is neither pus nor deposit, but a loosening of the tooth. A few months ago I made the remark that I believed I could cure as many cases of pyorrhœa as I could of caries of the teeth. I did not think it was any more incurable. I used the term in its generic sense. I do not think I would change that assertion. Regarding the uric acid theory, uric acid is a constituent of the normal urine. It is an element of destructive metabolism. There are cases without number of rheumatism and gout where the urinary analysis shows no uric acid. Again, we have many cases of pyorrhœa where there is no gout in the system. I kept a record of these cases for some time, and I found that those who said they had had rheumatism constituted only about forty per cent. I cannot see why this theory was ever taken up, as there seems to be no substantial basis for it. I have seen patients undergoing a constitutional treatment for months and continually getting worse recover in three or four treatments by instrumentation. The question of removing the tartar is most important. Years ago I read a paper before the New Jersey State Dental Society in which I stated that if a case of pyorrhœa alveolaris develops in your practice it is your fault and not the patient's. The removal of the tartar is a very difficult operation, as can be understood by taking these same teeth out of the mouth and endeavoring to do it. It is surprising what an amount of force is required. Dr. Eames's practical paper has given us a great deal of assistance. The instruments and the syringe are both practical. The syringe is most excellent. His methods are absolutely right. I am very glad to have had the pleasure of listening to him, and hope we may have the pleasure of hearing further from him. There is one point in the treatment which has been overlooked, and that is the bracing of very loose teeth to adjacent teeth. This I have accomplished quite successfully, keeping teeth in the mouth for years that would have otherwise been lost.

I wish to present some instruments for removing tartar. A feature of the instruments is their aluminum handles, insuring lightness.

*Dr. Charles O. Kimball.*—There is very little I want to say. I want to express, in behalf of the Committee, our very sincere thanks to Dr. Eames for his admirable paper. I cannot say too much for the intense practicality of it. Although he has gone sufficiently into the causes to make this reasonably clear, he has given us specially the practical side of the question. It is just what the Committee hoped he would do. The further discussion has been helpful. May I add one word to emphasize something that Dr. Gillett has said. The cases that are most helpfully treated are not the cases where there is a great mass of tartar. The cases that require our constant watchfulness are the children and the young people, keeping the tartar off by frequent cleansing. My experience has been that this can be done better not with a sharp but with a dull instrument. Now, this may be heretical, but I find that I can feel better with an extremely fine instrument, but one which has had the extreme edge slightly taken off. I can pass the instrument over a tooth and tell better whether I am working at a piece of tartar or a nodule on the tooth. The second thing to emphasize is what Dr. Gillett has said about small instruments. These beautiful instruments which Dr. Allan has shown us are undoubtedly very useful in bad cases, but in the case of a boy or girl where we have simply to remove a slight line under the gum I find I can do it well with but one instrument. An examination instrument with a blade one-eighth inch long and turned at nearly a right angle, about as large as a small cambric needle, flattened and with slightly rounded edges, I can pass under the free edge of the gum and on all sides of a tooth without starting a single drop of blood. Of course, the color of the gum will assist somewhat, but, after all, our main dependence, in my judgment, is in the educated touch.

*Dr. R. H. M. Dawbarn.*—I rise to ask if any gentleman here has used in this suppurative condition what the surgeons are now using a great deal, and that is "glutol"? I think it very well worth trying, especially for cleansing out pockets after the tartar has been removed. Glutol, you will remember, is made by exposing sheets of gelatin to formalin vapor. The formalinized gelatin is then ground down to a fine powder. The solvent action of the living tissue dissolves the gelatin, liberating the formalin, which is the antiseptic factor. This powder is of great value in controlling microbic activity in infected surfaces elsewhere, and should be here, too. It is entirely harmless to the patient, even if swallowed in large amount.

*Dr. Eames.*—I would like to ask Dr. Dawbarn if there is any reason why it would not be desirable to mix glutol with vaseline and use it in the syringe as suggested?

*Dr. Dawbarn.*—I can see no reason why it could not be used in this manner.

*Dr. Eames.*—Mention is occasionally made of serumal deposits on roots of teeth, from which there is no outlet to the margin of the gum. This seems to me to be an unscientific theory, for if this deposit is as irritating to the surrounding tissues as it is generally supposed to be, the resulting products of inflammation must find a way out, and the sinus found if a careful search is made. In regard to the cutting edge of scalers, it seems to me that these edges should be very sharp. This condition necessitates greater delicacy in handling, but the sense of touch should be sufficiently acute to prevent any damage being done by the removal of normal tissue. The advantages of thorough removal and of accomplishing the object with less force are quite apparent.

My feeling in regard to assistants is, that they may be capable of helping very much more than they ever have in the past, and that the patients themselves may do very much more towards the prophylactic home treatment and care of their mouths than they have hitherto done. We have yet to fully appreciate the importance of instructing patients in the care of their mouths and we have yet to learn how much a patient is capable of doing in the care of his own mouth.

I thank you, gentlemen, for your very kind courtesy in listening to me.

*Dr. Kimball.*—On behalf of the Executive Committee, I move that the thanks of the Institute be extended to Dr. Eames for coming here to-night and giving us this valuable paper. Carried.

Adjourned.

FRED. L. BOGUE, M.D., D.D.S.,
*Editor The New York Institute of Stomatology.*

